# Communication skills and raising awareness in clinical practice: an Italian experience

**DOI:** 10.3332/ecancer.2009.135

**Published:** 2009-02-11

**Authors:** S Liptrott, F Peccatori, A Cocquio, G Martinelli

**Affiliations:** 1 Division of Haemato-Oncology, European Institute of Oncology, 435 via Ripamonti, 20141 Milan, Italy; 2 Supportive Therapy and Palliative Care Unit, European Institute of Oncology, 435 via Ripamonti, 20141 Milan, Italy

## Abstract

Following reflection by a member of the healthcare team relating to a particularly difficult situation where communication between the healthcare professional, patient and family was felt to be challenging, there was a general consensus of interest in how we communicate, best practice methods and training opportunities. In order to look at the communication practice, skills and training within the department, it was felt best to identify how the team felt about their own communications skills as a baseline for development of this area of practice.

## Introduction

It is well documented that communication is an essential component of health-care, helping patients and their families to manage acute and chronic illnesses and improve the quality of their lives [1–3]—particularly important with people who face a frightening diagnosis and uncertain future for themselves or someone close to them [4].

Patients and carers place high value on face-to-face communication with health-care professionals, who can engage on an emotional level, listening and assessing patients’ information needs and providing information with clarity and sympathy [7]. This is especially relevant when dealing with patients with a cancer diagnosis, who may be facing difficult or complex decisions about treatment options. Specific communication needs at different stages must also be considered—diagnosis, treatment, recurrence, palliative and terminal care [5]. It is suggested that good communication is a pre-requisite for enabling patients and carers to make informed decision about care [8]. This is especially reflected in the haemato-oncology environment, where treatment decisions such as opting for haematopoietic stem cell transplant with its potential short-term and long-term complications needs to be discussed fully with patients in order to ensure realistic goals of treatment are understood.

Referring to patients with cancer, Maguire [6] suggests effective communication can make a great difference to the quality of life of patients throughout the whole cancer ‘trajectory’. The significance of good communication skills is reported within the literature [8,9]; however, despite the importance to both patients and the health-care team, research has indicated dissatisfaction with hospital staff’s communication skills [10] with one study identifying only 57% of patients are satisfied with health-care professionals’ listening skills, explanations and respect for their opinions [11].

In working with patients and families affected by cancer, the medical and nursing team within oncology are exposed to communication interactions with patients and families in all phases of the cancer ‘trajectory’. In a busy ward, it can be difficult to dedicate an appropriate level of attention to the needs of patients and carers; however in this setting, clarity of communication is vital if patients are to participate in decision-making process. Although recommendations for demonstration of effective communication skills are a pre-condition of qualification for health-care professionals working with cancer patients in the United Kingdom, communication skills training is not currently mandatory in Italy.

## Background to the project

Following reflections by a member of the health-care team relating to a particularly difficult situation where communication between the health-care professional, patient and family was felt to be challenging, there was a general consensus of interest in how we communicate, best practice methods and training opportunities.

In order to look at the communication practice, skills and training within the department, it was felt best to identify how the team felt about their own communications skills as a baseline for development of this area of practice.

## Aims

The aims of this piece of research were to investigate the following questions:

- What are the perceptions of personal communication skills in the department?
- What are the particular problems or challenging situations faced?
- Do staff feel they have particular strengths or weaknesses in this area of their practice?
- Are there areas of communication skills that staff felt needed development?
- Are there differences in perceptions between medical and nursing teams or in their length of clinical practice?

## Methodology

Within the department, a variety of communication scenarios take place with patients, families and other health-care professionals, focusing on the care of patients newly diagnosed, those undergoing intensive treatment with stem cell transplantation and those receiving palliative and terminal care. To capture the experiences of the doctors and nurses working with these patients and families, a survey method was chosen as a simple way to collect a lot of data in a short time period. Furthermore, it is anonymous, cost-effective and easy to complete [12]. A questionnaire was designed incorporating both closed and open ended questions—this combination was chosen to elicit both specific information and to give the respondent the possibility of providing a fuller perspective on topics felt to be of importance. One question used a visual analogue scale (VAS) to depict the respondents’ global view of their effectiveness in communicating.

The questionnaire was constructed using research-based literature where key aspects of the communication process were highlighted [8, 14–18]. This questionnaire was sent to senior members of the medical and nursing teams to ensure that all key areas of communication had been incorporated and, following this review, one additional question was added to the questionnaire. Although this was not a standardized tool, the aim was to demonstrate content validity by a systematic examination of the questionnaire content to determine whether it covers a representative sample of the behaviour domain to be measured [13].

The questionnaire was sent out to the 32 members of staff (14 doctors and 18 nurses) working within the haematology department of a cancer-specific hospital in Italy. This included medical staff rotating between inpatient, outpatient and day hospital settings, and all nursing staff working within the inpatient unit. The opportunity for anonymous return was given.

The questionnaire incorporated:

- general demographic information related to type of health-care professional and the number of years of clinical practice;
- communication practice—who is, and who should be present and the environment where ‘communication’ takes place;
- information giving—formats available and staff preferences, time allowed to give information;
- situations felt to be significant or difficult to handle;
- previous training and interest in training.

## Results

### Demographic data

Of the 32 questionnaires that were sent out to medical and nursing staff, 14 (43%) responded (five nurses and nine doctors). Table 1 shows a distribution of years in oncology practice between nurse and medical staff responders.

### Giving information about disease, prognosis and treatment to patients and relatives

Staff were asked about their role in giving information about disease, prognosis and treatment to patients and relatives. Of the respondents, 79% (*n*=11) acknowledged that they had a role in information giving, while three respondents, all nurses, said they did not give this information.

Nine of the respondents (63%) felt only the doctor should communicate diagnosis/prognosis and patients’ conditions—eight of these were doctors. The main reasons for this choice was that the competency and knowledge of the doctor was more detailed.

When communicating diagnosis/prognosis and patients conditions, eight of the responders (56%) felt the patient, doctor and nurse should be present. Ensuring consistency of information and avoiding jargon were reasons given for this multidisciplinary approach. Only three responders identified that it should be the patients’ choice; however in a later question, the presence of friends/relatives at this type of communication was felt to be the patients’ choice in 50% of the responses

In preparing for communicating diagnosis/prognosis/condition, the respondents said that they used various methods, including history, laboratory and clinical data, and discussion with colleagues before the communication process. Staff were also asked if they employed particular methods to prepare the patients for the communication/consultation. Methods of patient preparation were varied and included giving advanced warning about the meeting, progressive preparation, reassurance and distraction, and environmental features.

The global self-evaluation, using a visual analogue scale of overall effectiveness in communicating to patients and relatives, where 0 was ‘not at all effective’ and 10 was ‘very effective’, gave a mean evaluation of 7.13.

### Environment

Staff were asked to identify important environmental factors when communicating this type of information regarding disease, prognosis or treatment decisions. Key factors were seen to be ‘a room without another patient/without interruptions (63%) and dedicated time. Over half of the respondents (56%) said they did not have access to this type of environment

### Information and questions

Staff were asked about the actual information given during the communication process. Forty-two per cent of responders felt all information available should be given, and the remaining 56% felt that the amount of information provided should actually reflect as much as the patient wants. The format preferred by 77% responders was a combination of both written and verbal.

All responders said they allowed patients time for questions during this discussion, and eight responders (56%) said the time required for this type of communication was ‘all the time necessary’. The remaining 44% identified a time frame ranging from one to two hours. No significant difference was seen between the groups regarding their responses when stratified in terms of profession and years in practice.

All but one of the respondents said they gave patients or carers the opportunity to re-discuss issues again, and only five responders (35%) felt they had the opportunity to leave the patient in a room/appropriate area with another health professional if required. All but one responder said they informed other colleagues when they had given significant news—the other respondent left this section blank.

### Difficult scenarios

Staff were asked to identify any particularly difficult scenarios where they felt their communication skills were challenged. Only five responders replied to this question (Table 2), and a variety of themes were identified, including competency, the doctor–patient relationship, similar personal situations, dealing with family members and aggression.

### Communication skills training

Of the responders, seven (49%) said they had never received any formal communication skills training, five (36%) had received some form of education/training and a further two staff members did not answer this question. Nine responders (63%) felt they could benefit from communication skills training.

## Discussion

From the results of the questionnaire some key points were identified.

The perception of overall effectiveness of communication skills of the responders regarding communication of diagnosis, prognosis and condition was high (mean 7.13, where 10 was the maximum). Practical aspects of giving information about disease, prognosis and treatment to patients and relatives were highlighted in terms of the importance of environment type and availability, having the personnel necessary for this type of communication, and patient choice. The need for supportive written information reflects findings by Smith [19] in a patient survey, where 86.8% of respondents preferred to have information written and/or discussion with health-care professionals. Environmental concerns were identified by many responders; however, the majority did not feel they had access to an ‘ideal’ type of environment. The difficult communication scenarios identified by respondents reflects those reported in the literature [16], and these can be used to form the basis for future communication skills development for the team.

The responders identified different methods and techniques for communication; however, this appeared independent of medical or nursing background, and time in clinical practice. Cantwell and Ramirez [20] suggest this is a skill to be learnt—not only by experience. A lack of formal training in communication skills was identified; however, the majority of responders felt they could benefit from some formal training. This is a feeling reflected in the literature, where professionals may feel inadequately trained in some communication aspects—such as exploring uncertainty and discussing end-of-life issues [8]. One study of medical and nursing students participating in a breaking bad news workshop showed an improved personal perception of communication skills after course participation [26] and effective training has been shown to increase competence [21], which is a problem recognized by this group of respondents.

The overall, the views of the team showed no great difference between years of practice or profession. The differences between doctor and nurse responses in terms of numbers may be influenced by the lower number of nurse responses, but also the variability in communication tasks and perceived difficulty of the tasks should not be underestimated.

## Limitations

The proportion of responders was only 43%, and although this percentage is acceptable, the total number remains low and those motivated to respond to the questionnaire may be more interested in this field—potentially biasing the results. The used of a non-standardized questionnaire also limits the comparability of this study, and tests for validity and reliability of the questionnaire were not rigorously performed. It was felt in this situation, however, that the use of a tool to reflect the clinical situation and provide less structured, but more qualitative approach, was the most appropriate to elicit what could be rather emotional and difficult responses.

## Conclusion

It is recognized that this was a small ‘in-house’ project to identify a baseline for staff, and that its subsequent application to different institutions may be limited; however, it has been fundamental in raising awareness of the issue of communication and encouragement of reflection on daily practice in order to give some direction for service development, in an area where the need for communication skills training is not currently seen as mandatory but may be beneficial not only for patients but staff well-being. This has been an interesting project to identify communication practice, skills and training within a busy haemato-oncology department by means of a multidisciplinary evaluation. The presentation of this project and its results have provided an open forum for discussion regarding self-perception of skills, instruments and training needs for staff in providing the best possible care for patients. This project has been presented to the Communication Working Group of the hospital and has been taken forward by another department hoping to complete the same questionnaire and identify some comparative results.

## Implications for practice

This questionnaire has provided the basis for our work in process and has identified areas for our future practice development including:

- Training—discussions with the Psychology Unit of the hospital are in progress for a more formal event within the haemato-oncology department involving structured communication/role play, etc. Implementation of a skills training programme, such as those described by Fallowfield *et al* [22] and Razavi *et al* [23], has demonstrated positive effects on the behaviours of experienced nurses and doctors in cancer care suggesting such training could be beneficial.
- Environment—the possibility of having a dedicated area for communicating significant news is an issue to be addressed.
- Personnel involved in this communication process—ensuring all people present. Teamwork is identified as an important feature of good clinical practice [24,25] and, in situations where patients and carers may ask nurses to clarify or expand on issues dealt with by medical staff, nurses should be prepared to address
- information needs within their competency.
- Development of written information to support verbal information given.

Future service development should also include evaluation of patients’ perceptions of the communication process and of any service improvement initiatives.

## Figures and Tables

**Table 1:**
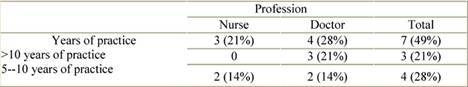
Respondents demographic data

**Table 2:**
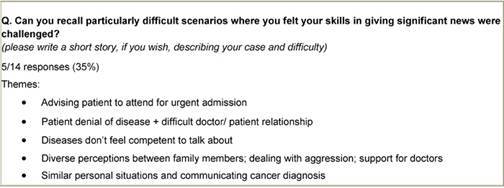
Difficult scenarios
